# Kinderreanimation mit anhaltendem Kammerflimmern – ein Fall für ein mechanisches Reanimationsgerät?

**DOI:** 10.1007/s00101-023-01275-3

**Published:** 2023-04-25

**Authors:** M. Irrgang, S. Beckers, M. Felzen, G. Schälte, R. Rossaint, H. Schröder

**Affiliations:** 1 Klinik für Anästhesiologie, Medizinische Fakultät RWTH Aachen, Uniklinik RWTH Aachen, Pauwelsstr. 30, 52074 Aachen, Deutschland; 2 Aachener Institut für Rettungsmedizin und zivile Sicherheit, Uniklinik RWTH Aachen & Stadt Aachen, Aachen, Deutschland

## Einleitung

Mechanische Reanimationsgeräte (mCPR-Geräte) versprechen eine hochwertige Thoraxkompression und eine Erleichterung der Reanimation für das beteiligte Rettungsdienstpersonal. Mechanische Reanimationsgeräte werden mittlerweile deutschlandweit prä- wie innerklinisch eingesetzt, um eine sichere und effektive Thoraxkompression unter schwierigen oder für das Personal gefährlichen Situationen zu gewährleisten. Jedoch existieren nur wenige Daten und kaum Empfehlungen für den Einsatz in der Reanimation von Kindern. Dies mag einerseits den vergleichsweise niedrigen Inzidenzen von prä- (3–8/100.000 Kinder und Jahr [4, 10]) und innerklinischen (zwischen 0,7 % aller stationär aufgenommenen und 5,5 % der intensivmedizinisch behandelten Kinder [15]) Kinderreanimationen geschuldet sein. Diesen steht eine Gesamtinzidenz des Herz-Kreislauf-Stillstands von 135,8/100.000 Einwohner prähospital sowie 1,6/1000 stationären Fällen (bezogen auf das Jahr 2021 [5, 12]) gegenüber. Andererseits existiert Stand Dezember 2022 mit dem Corpuls CPR® (GS Elektromedizinische Geräte G. Stemple GmbH, Kaufering, Deutschland) nur ein mCPR-Gerät mit Zulassung für Kinder ab 8 Jahren, sodass die Anwendung hierdurch zusätzlich limitiert ist [2]. Neben geringerer Praxiserfahrung der Anwender, v. a. in der Präklinik, ergibt sich hieraus auch eine schlechtere Datenlage mit Blick auf Indikation und Anwendung von mCPR-Geräten, von einzelnen Fallberichten abgesehen [14].

Die folgende Fallvorstellung berichtet vom Einsatz eines mCPR-Geräts beim Kind und zeigt, dass der Einsatz unter bestimmten Voraussetzungen sinnvoll sein kann.

## Falldarstellung

Ende September 2022 wurden ein Rettungswagen (RTW) und ein Notarzteinsatzfahrzeug (NEF) mit der Meldung „bewusstloses Kind nach Sturz“ in die Aachener Innenstadt alarmiert. Der Notruf erreichte die Leitstelle weitergeleitet von der Polizei, sodass Rückfragen an den Anrufer nicht möglich waren. Dadurch fehlten die Optionen zur direkten Alarmierung auf „Kinderreanimation“ sowie zu Ersthelferalarmierung und Anleitung zur telefonischen Reanimation.

Bereits 2 min nach Alarm traf der Rettungswagen an der Einsatzstelle ein (Tab. 1), wo ein Junge, auf dem Gehweg liegend, durch den Vater laienreanimiert wurde. Es erfolgten die Übernahme der Thoraxkompressionen durch die RTW-Besatzung und die Vorbereitung des Atemwegsmanagements. Die zunächst angelegten Defibrillatorklebeelektroden in Erwachsenengröße wurden nach den Gewichtsangaben des Vaters auf Kinderelektroden gewechselt.

**Table 1:** 

*Notrufeingang*	16:27 Uhr, Integrierte Leitstelle Stadt Aachen		
*Einsatzmittel*	*Alarm*	*Fahrzeit bis Einsatzstelle*	*Verweildauer Einsatzstelle*
RTW	16:29 Uhr	2 min	25 min
NEF	16:29 Uhr	5 min	24 min
LNÄ NEF, HLF	16:37 Uhr	5 min	17 min
*Übergabe*	17:05 Uhr, Schockraum Uniklinik RWTH Aachen		

*RTW* Rettungswagen, *NEF* Notarzteinsatzfahrzeug, *LNÄ NEF* Leitende Notärztin mit NEF, *HLF* Hilfeleistungslöschfahrzeug

Bei Eintreffen des NEF, 3 min nach dem RTW, wurde das Meldebild nach unmittelbarer Rückmeldung durch den Notarzt auf „Kinderreanimation“ erhöht. Es erfolgte entsprechend der lokalen Alarm- und Ausrückeordnung der Nachalarm der diensthabenden Leitenden Notärztin (LNÄ) sowie eines Hilfeleistungslöschfahrzeugs (HLF) der Berufsfeuerwehr Aachen zur personellen und logistischen Unterstützung.

**Figure 1:**
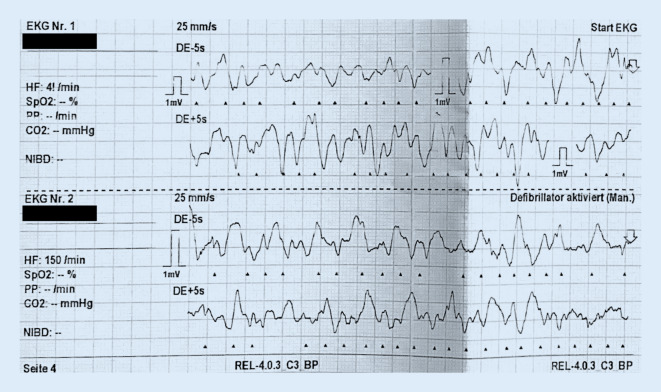

Das Atemwegsmanagement wurde durch den Notarzt an einen zweiten Anästhesisten im Einsatzpraktikum delegiert. Unter Beutel-Maske-Beatmung besserte sich die initiale Zyanose rasch. In der ersten Rhythmusanalyse bot sich das Bild eines groben Kammerflimmerns (Abb. 1), sodass unmittelbar eine Defibrillation mit 4 J/kgKG durchgeführt und die Reanimation nach pädiatrischem Advanced Life Support (PALS) fortgesetzt wurde. Es erfolgten die Einlage einer Larynxmaske Supreme™ #2.5 (The Laryngeal Mask Company Limited, Mahé, Seychellen) sowie Etablierung eines i.o.-Zugangs auf dem rechten Tibiaplateau.

## Anamnese

Der 10-jährige Junge war laut Vater ohne Vorzeichen plötzlich kollabiert und hatte eine unnormale Atmung, sodass der Vater sofort die Herzdruckmassage begann. Ein vorheriges Trauma oder Bolusereignis wurde verneint. Es bestanden keine Vorerkrankungen, lediglich eine Nussallergie ohne Allergenkontakt. Konsultationen eines Pulmonologen bei vermehrten Atemwegsinfekten in den letzten Monaten blieben ohne Befund.

## Verdachtsdiagnose

Bei Berücksichtigung der reversiblen Ursachen (4H/HITS) und sichergestellter Oxygenierung (etCO_2_ ≈ 30 mm Hg) wurde bei persistierendem Kammerflimmern als wahrscheinlichste Ursache ein kardiales Ereignis, am ehesten bei unbekannter Herzerkrankung wie einer Kardiomyopathie (Inzidenz 1–2/100.000 Kinder [3, 6]), postuliert, obwohl klassische Symptome wie eingeschränkte Belastbarkeit, Dyspnoe, Schwindel oder Herzrhythmusstörungen durch den Vater nicht berichtet wurden.

## Prähospitaler Verlauf

Unter suffizienten PALS-Maßnahmen wurden Abwehrbewegungen des Jungen und ein Pressen gegen die Larynxmaske bemerkt, in den folgenden Rhythmusanalysen zeigten sich jedoch persistierendes Kammerflimmern und fehlende zentrale Pulse.

**Figure 2:**
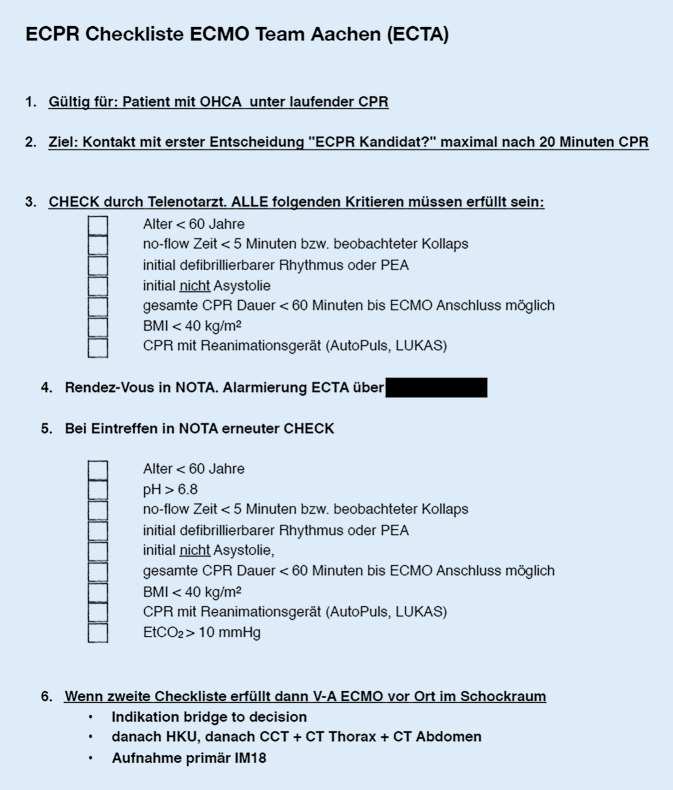

Bei Erfüllung aller Kriterien der lokal etablierten Checkliste (Abb. 2) zur extrakorporalen Reanimation (eCPR) in der Uniklinik RWTH Aachen (UKA) und vermuteter kardialer Ursache erfolgte rund 6 min nach Eintreffen des Notarztes die Voranmeldung beim Triagearzt der Interdisziplinären Notaufnahme der UKA zur Evaluation einer eCPR und weiterführenden Therapie. Nach dem dritten frustranen Schock wurden leitlinienkonform 10 µg/kgKG Adrenalin sowie 5 mg/kgKG Amiodaron i.o. verabreicht.

Durch den Fahrer des NEF waren bereits die Transportvorbereitungen angelaufen. Die Fahrtrage stand mit einsatzbereitem mCPR-Gerät (Corpuls CPR®) in unmittelbarer Nähe zur Einsatzstelle.

Die eintreffende LNÄ nahm nach kurzem Briefing eine koordinierende und unterstützende Rolle ein. Die Besatzung des HLF wurde unterstützend durch Übernahme der Herzdruckmassage, Errichten eines Sichtschutzes und Betreuung der Angehörigen tätig. Nach Umlagerung auf die Fahrtrage erfolgten die korrekte Platzierung, der Start des mCPR-Gerätes und die Verbringung in den RTW, wo die Defibrillationselektroden zur Eskalation der Therapie auf 6J/kgKG auf Erwachsenen-Elektroden gewechselt wurden. Die endotracheale Intubation im RTW gelang bei unter Reanimation vorhandenen Abwehrbewegungen des Kindes nicht, trotz Cormack-Lehane-Score I–II. Aufgrund der Nähe zur UKA wurde einsatztaktisch auf eine Narkoseinduktion zur endotrachealen Atemwegssicherung verzichtet, erneut die Larynxmaske Supreme™ #2,5 etabliert und die Beatmung hiermit mit intermittierender Absaugung des Magens über die einliegende Magensonde komplikationslos fortgeführt (etCO_2_ 29–41 mm Hg). Das Kind erhielt gewichtsadaptiert 0,1 mg Fentanyl zur Abschirmung. Parallel wurde der Transport begonnen.

## Stationärer Verlauf

38 min nach der Alarmierung wurde das Kind unter laufender mCPR im Schockraum der UKA eingeliefert. Vor dem Ausladen des Patienten aus dem RTW erfolgte die letzte Rhythmuskontrolle durch das Rettungsteam, welches bei weiterhin persistierendem Kammerflimmern erneut defibrillierte. Nach Übergabe im Schockraum erfolgten die sofortige Anlage einer venoarteriellen ECMO sowie die Notfallnarkose und endotracheale Intubation. Zu diesem Zeitpunkt war eine pulslose elektrische Aktivität (PEA) der vorherrschende Herzrhythmus. In der Primärdiagnostik zeigte sich eine linksbetonte hypertrophe Kardiomyopathie mit Septumverdickung und ausgedehnten linksventrikulären, septal-betonten Infarktarealen bei unauffälligen Koronararterien.

Nach der Erstdiagnose einer hypertrophen Kardiomyopathie erholte sich die Pumpfunktion des Herzens unter Entlastung durch die ECMO und intensivmedizinischer Therapie. In den ersten 24 h ging die PEA in einen Sinusrhythmus mit Auswurf über, und die ECMO wurde eine Woche nach dem Ereignis explantiert. Es erfolgte die prophylaktische Anlage eines Ein-Kammer-ICD.

22 Tage nach der Aufnahme wurde das Kind in leicht reduziertem Allgemeinzustand, jedoch ohne neurologische Folgen entlassen und befindet sich seitdem in engmaschiger kinderkardiologischer Kontrolle. Ein möglicher kausaler Gendefekt wurde in der Folge identifiziert.

## Diskussion

### Mechanische Reanimationshilfe beim Kind?

mCPR-Geräte sind in der Therapie des Herz-Kreislauf-Stillstands von Erwachsenen nicht unumstritten. Zwar sind sie in der Lage, eine Thoraxkompression mit vorgegebener Tiefe und Frequenz mit gleichbleibender Qualität durchzuführen, ohne dass Einbußen durch Ermüdung oder Qualifikation der Helfer oder durch Störfaktoren wie den Transport in Kauf genommen werden müssen.

In vielen Studien konnten allerdings lediglich eine Gleichwertigkeit, jedoch keine Überlegenheit zu einer manuellen Thoraxkompression und keine Verbesserung des Outcomes nachgewiesen werden [7–9]. Zudem ist der schnelle und erfolgreiche Anschluss eines mCPR-Geräts abhängig von äußeren Faktoren und der Routine der Anwender im Umgang mit dem Gerät.

Mit Blick auf die Datenlage empfehlen daher die Reanimationsleitlinien des European Resuscitation Council aus dem Jahr 2021 keinen routinemäßigen Einsatz. Stattdessen soll der Einsatz auf Situationen beschränkt werden, in denen eine manuelle Thoraxkompression entweder für den Patienten oder das durchführende Personal nicht effektiv oder nicht sicher durchgeführt werden kann [13]. Unter Berücksichtigung der geringen Inzidenz von Kinderreanimationen überrascht es daher kaum, dass zum Einsatz von mCPR-Geräten bei Kindern, abgesehen von einzelnen Fallberichten, kaum Erfahrungen existieren [14].

Dementsprechend gibt es keine Empfehlungen zum Einsatz von mCPR-Geräten bei Kindern. Als Ergebnis eines Expertenworkshops im Jahr 2021 zu ethischen Aspekten der mCPR bei Kindern wurde jedoch davor gewarnt, dass der Einsatz von mCPR nicht zu einer Verschiebung der Entscheidungsfindung über eine Beendigung der Reanimation in die Klinik führen dürfe [17]. Dies ist analog zu den Empfehlungen bei Erwachsenen zu betrachten.

Die in Aachen verwendete Checkliste ist nicht explizit auf Erwachsene beschränkt (jedoch primär für diese entwickelt) und dient den NotärztInnen zur Unterstützung in der Entscheidungsfindung, ob ein Transport unter mCPR in die UKA erfolgt. Bei dem Kind wurde bei suffizienter Laienreanimation ohne Latenz, persistierendem Kammerflimmern und bei am ehesten kardialer Ursache die Indikation zur mCPR durch die involvierten NotärztInnen großzügig gestellt. Auch, weil das stets suffiziente etCO_2_ und die Spontanbewegungen des Kindes als Anhalt für ein potenziell gutes Outcome gewertet wurden.

Zudem stand, mangels prähospitaler Therapieoptionen, der schnelle, sichere Transport zum 4 km entfernten Maximalversorger zur zielgerichteten Therapie und der Option einer eCPR im Vordergrund. Eine manuelle Reanimation hätte eine deutliche Reduktion der Transportgeschwindigkeit bedingt, um die Qualität der Thoraxkompressionen sowie die Sicherheit des Personals zu gewährleisten.

In einer Analyse von Anton-Martin et al. wurden 73 Kinder, bei denen eine eCPR-Therapie durchgeführt wurde, nachverfolgt. 43,8 % verließen das Krankenhaus lebend, 75 % davon ohne oder mit nur leichten neurologischen Beeinträchtigungen [1].

Die eCPR wird bei Kindern nach ERC-Leitlinie 2021 nicht explizit empfohlen. Lediglich beim „inhouse cardiac arrest“, bei dem konventionelle PALS-Maßnahmen nicht sofort zu einem wiedereinsetzenden Spontankreislauf führen, sollte die eCPR in Betracht gezogen werden [16].

### Atemwegssicherung unter mCPR?

Um den Transport nicht weiter zu verzögern, wurde nach frustranem Intubationsversuch in Abweichung von der Empfehlung der aktuellen ERC-Leitlinie 2021 auf eine endotracheale Intubation verzichtet, und bei dichter Larynxmaske ab diesem Zeitpunkt eine kontinuierliche Herzdruckmassage durchgeführt und das Kind über die Larynxmaske unter permanenter Beobachtung manuell beatmet. Wäre keine ausreichende Dichtigkeit der Larynxmaske erreicht worden, hätte als Alternative eine intermittierende Thoraxkompression mit abwechselnder Beatmung zur Verfügung gestanden. Weder eine Überblähung des Magens noch eine Aspiration konnte nach der Aufnahme nachgewiesen werden, sodass der Einsatz einer Larynxmaske Supreme™ mit Entlastungssonde rückblickend als effizient beurteilt werden kann.

### Intervalle der Rhythmuskontrolle

In der nachträglichen Analyse des Einsatzes fiel eine Verlängerung der Intervalle zur Rhythmuskontrolle und Defibrillation auf, im Schnitt 288 s nach Etablierung der mCPR.

In diesem Kontext stachen zwei Situationen heraus:

Trotz Briefing des Teams und Umsetzung direkt nach der Defibrillation waren Umlagerung, Anschluss und Start des mCPR-Geräts für das Team nicht innerhalb von 2 min zu bewältigen, sodass die nächste Rhythmusanalyse und Defibrillation erst nach 213 s erfolgten.

Eine weitere Verzögerung entstand durch den frustranen Intubationsversuch und gleichzeitigen erneuten Wechsel auf Erwachsenendefibrillationselektroden, wodurch eine Pause von 468 s entstand. Auch unter diesem Aspekt erscheint nachträglich der Einsatz einer Larynxmaske Supreme™ als probat.

Unabhängig davon wurde retrospektiv von mehreren Beteiligten aufgrund der fehlenden manuellen Thoraxkompressionen eine Distanzierung zur Reanimation beschrieben, mit Verlust des Zeitgefühls. Auch wenn hochqualitative Thoraxkompressionen durch den Einsatz der mCPR während des gesamten Transports sichergestellt wurden, so muss kritisch angemerkt werden, dass die zeitliche Koordination nicht aus den Augen verloren werden darf. Hier bedarf es zukünftiger Registerauswertungen.

### LNÄ zur Kinderreanimation?

Bei der Einbindung der LNÄ-Funktion in die lokale Alarm- und Ausrückeordnung steht weniger die eigentliche Funktion im Fokus als vielmehr die zügige Verfügbarkeit einer zweiten, erfahrenen Notärztin. Obwohl bedarfsabhängig auf die fachliche Expertise der LNÄ zurückgegriffen werden kann, liegt der Schwerpunkt vielmehr in der Einsatzunterstützung in den Bereichen Einsatztaktik, Organisation und Kommunikation. Die Koordination und das Management der Reanimation verbleiben, wenn möglich, bei der ersteintreffenden Notärztin. Nach dem Einsatzende gehören auch die Organisation einer fachlichen Nachbesprechung und der psychosozialen Unterstützung sowie die bedarfsabhängige Nachführung von Personalressourcen zur Auslösung zu den Aufgaben der lokal eingebundenen LNÄ.

## Fazit für die Praxis

- mCPR kann – unter Beachtung von Indikation, Patientenalter und Gerätezulassung – bei Kindern erfolgreich eingesetzt werden, um einen gefahrlosen Transport unter hochqualitativer Thoraxkompression zu gewährleisten.
- Gleichfalls sollte mCPR nur mit klarer Zielsetzung erfolgen und nicht die Entscheidung zur Beendigung der Reanimation in die Klinik verlagern.
- Der Einsatz von mCPR kann zu zeitlichen Abweichungen im Reanimationsalgorithmus führen. Maßnahmen sollten daher geplant und koordiniert werden, um Verzögerungen zu vermeiden. Die Benennung eines Zeitnehmers kann hilfreich sein.
- Nach ERC-Leitlinie 2021 können bei endotracheal intubierten Kindern „die Beatmungen asynchron und die Thoraxkompressionen kontinuierlich erfolgen“. Obwohl supraglottische Atemweg-Devices keinen vollständigen Aspirationsschutz bieten, erscheinen der Verzicht auf eine endotracheale Intubation und die Beatmung via Larynxmaske Supreme™ mit Entlastung des Magens per Magensonde bei kurzen Transportwegen durch den möglichen Zeitgewinn legitim. Kontinuierliche Überwachung ist dabei obligat, um bei Komplikationen auf abwechselnde Kompression und Beatmung zu wechseln.
- Die frühestmögliche Alarmierung einer weiteren Notärztin (ggf. LNÄ) erscheint sinnvoll, um vor Ort Einsatztaktik, Schnittstellenkommunikation und Organisation zu übernehmen und im Bedarfsfall als Fachexperte zu unterstützen.
